# Genomic insights into the spread and evolution of insecticide resistance variants in *Anopheles gambiae* s.l. from Burkina Faso

**DOI:** 10.1038/s41598-026-45950-y

**Published:** 2026-04-01

**Authors:** Mahamadi Kientega, Honorine Kaboré, Grégoire Sawadogo, Tin-Yu J. Hui, Nouhoun Traoré, Abdoul-Azize A. Millogo, Hamidou Maiga, Alistair Miles, Chris S. Clarkson, Abdoulaye Diabaté

**Affiliations:** 1 Institut de Recherche en Sciences de la Santé (IRSS), 01 BP 545 Bobo- Dioulasso, Burkina Faso; 2 Vector Surveillance Programme, Genomic Surveillance Unit, Wellcome Sanger Institute, Hinxton, Cambridge, UK; 3 Université Nazi Boni, 01 BP 1091 Bobo-Dioulasso, Burkina Faso; 4 Department of Life Sciences, Imperial College London, Silwood Park, Ascot, SL5 7PY UK

**Keywords:** Insecticide, Resistance, Genomics, *An. gambiae* s.l., Malaria, Evolution, Genetics, Molecular biology

## Abstract

The intensive use of insecticide-based control tools has led to the rapid evolution of resistant phenotypes in malaria vector populations. Understanding the evolutionary processes underlying these resistances is essential to inform the development and deployment of effective control interventions. This study investigated the geographical spread and the genetic background of insecticide resistance variants in *Anopheles gambiae* s.l. in Burkina Faso. The study identified five pyrethroid-resistant mutations (*995F*,* 995S*,* 402L(g > t*,*c)*,* 1527T* and *1570Y)* at high frequencies. Six diplotype groups were identified, including novel combinations of the resistance-associated alleles (*995F*,* 402L(g > t*,*c)* and *1527T*), which formed new genotypes within *An. coluzzii* populations. These results suggest the emergence of new resistance genotypes in *An. coluzzii* that are not associated with 995F, probably due to recombination and gene flow events. Interestingly, strong linkage disequilibrium (*r*^*2*^ *= 0.821*) was observed between *1527T* and *402L(g > t)* compared to *1527T* and *402L(g > c)*. The PCA revealed three clusters of *An. coluzzii* populations, driven by *995F*,* 402L(g > t*,*c)* and *1527T*. Other insecticide resistance associated variants such as copy number variations and SNPs in the *Ace1* gene (*ace1-G280S*), cytochrome P450s, esterases and glutathione S-transferases were identified at high frequencies in the same mosquito populations, indicating the intensity and diversity of resistance mechanisms in the country. The study underscores the extent and spreads of insecticide resistance variants in Burkina Faso. It highlights the importance of genomic surveillance of malaria vectors to monitor and detect new resistance variants and to understand the evolutionary processes in vector populations.

**Supplementary Information:**

The online version contains supplementary material available at 10.1038/s41598-026-45950-y.

## Introduction

Sub-Saharan Africa has high malaria endemicity, with more than 90% of the cases and deaths reported worldwide occurring within the region^1^. Due to its tropical climate and environment, this region is home to the major malaria-carrying mosquitoes, including *An. gambiae* s.l. and *An. funestus*. In Burkina Faso, malaria is endemic, and the transmission patterns are modulated by the ecoclimatic and rainfall patterns, showing a high transmission intensity during the rainy season^2^. Malaria dynamics encompass a long and high transmission intensity over 6 months in the southern part of the country that is progressively shortened moving northerly due to the shortening of rainfall season^3^. Like other sub-Saharan countries, *An. gambiae* complex species remain the major malaria vectors in Burkina Faso. Three species of this complex, *An. arabiensis*,* An. coluzzii* and *An. gambiae* sensu stricto have been widely described in the country^4^. These species are living in sympatry with other vectors, including *An. funestus*, throughout the ecological zones of the country^5,6^.

In Burkina Faso, current malaria control interventions include several strategies encompassing vector control tools, antimalarial drugs, community and environmental management and a recent introduction of malaria vaccine (*RTS*,* S*) targeting the children of 5 to 17 months old^7^. Of these strategies, vector control is a key pillar of malaria prevention relying on insecticide treated nets (ITNs) and indoor residual spraying (IRS) to reduce mosquito density. The wide implementation of these tools has led to significant achievement over the past two decades in the reduction of malaria burden. These substantial achievements include a decline of malaria burden of over 50% between 2000 and 2015 with more than 68% attributed to the use of the insecticide treated nets^8^. However, the high adaptability of the African malaria vectors *An. gambiae* s.l. and *An. funestus* species group has reduced the ability of the insecticide-based tools to continue reducing malaria incidence through the development of resistance^9,10^. This situation causes serious threats to the long-term efficacy of the current malaria control tools in Africa as the malaria mosquitoes have widely developed resistance to most of the insecticide compounds used in public health. Several countries including Burkina Faso have adopted a plan for insecticide resistance management under the umbrella of WHO and its partners, including PMI, USAID, the malaria consortium, the Global Fund etc^11^. This plan includes the rotation and combination of different insecticide-based tools; the introduction of new insecticidal compounds with different modes of action, the use of synergist-treated tools, and encouraging the development of new and complementary tools through genetic engineering^12^. Although this plan appears sustainable, the successful integrated vector management (IVM) programs require a robust surveillance strategy to understand the vector dynamics and evolutionary patterns impacting the efficacy of control tools.

The recent advances in the *An. gambiae* genomics have provided deep insights into the evolutionary patterns and genetic makeup of these resistance mechanisms. Studies of genetic makeup within the gene encoding voltage-gated sodium channel (*Vgsc*) revealed the more complex molecular basis of the pyrethroid target site resistance in the *An. gambiae* s.l. populations in Africa^13^. The main *knock-down resistance* (kdr) mutations (i.e. *vgsc-L995F* and *vgsc-L995S*) are shown widespread among vector populations, at high frequencies in many African regions. Additional mutations (i.e. *vgsc-N1570Y*,* vgsc-V402L* and *vgsc-I1527T*) have been identified alongside both mutations within the *Vgsc* gene and were shown to contribute to the increase of the pyrethroid resistance^14,15^. It has long been thought that the kdr mutations have not emerged in the *An. funestus* populations. However, recent advanced genomic studies of this mosquito have identified two variants, the *L976F* and *P1842S*, within the *Vgsc* locus (*LOC125769886*,* 3RL:44105643–44156624*) and shown to be associated with the resistance to DDT^16^. Although no direct link between both mutations and pyrethroid resistance was observed, their emergence within the *Vgsc* gene could enhance the level of pyrethroid resistance that is already driven by detoxifying gene expression^17^ and the discovery of these mutations proved the high importance of genomic surveillance.

Recent studies also revealed the complex evolution of the malaria vectors in Burkina Faso, showing multiple changes in the genetic makeup of the insecticide-targeted genes within the *An. gambiae* s.l. populations^18,19^. These studies identified several variants including pyrethroid target-site, the organophosphate resistance variants and copy number variation in the detoxifying genes within the same mosquito populations^9,20^. Furthermore, the trends and evolution of these variants show a marked increase in the prevalence of resistance to the main insecticides used in malaria control. Although these findings inform the integrated vector management (IVM) programs in Burkina Faso, it is limited to the western part of the country^9^ and no comprehensive data on the spread and the prevalence of these variants across the country is available. Understanding the geographical spread and the evolutionary patterns underlying the genetic makeup of the insecticide resistance is essential to inform the global integrated vector management program. In this study, we investigated the geographical spread and the genetic background of insecticide resistance variants in Burkina Faso. The study leveraged whole genome sequencing data of wild *An. gambiae* s.l. from country-wide sampling to investigate the geographical distribution and the genetic background of the insecticide resistance variants in Burkina Faso.

## Results

### Pyrethroid target-site resistance

#### Genetic diversity

**Fig. 1:**
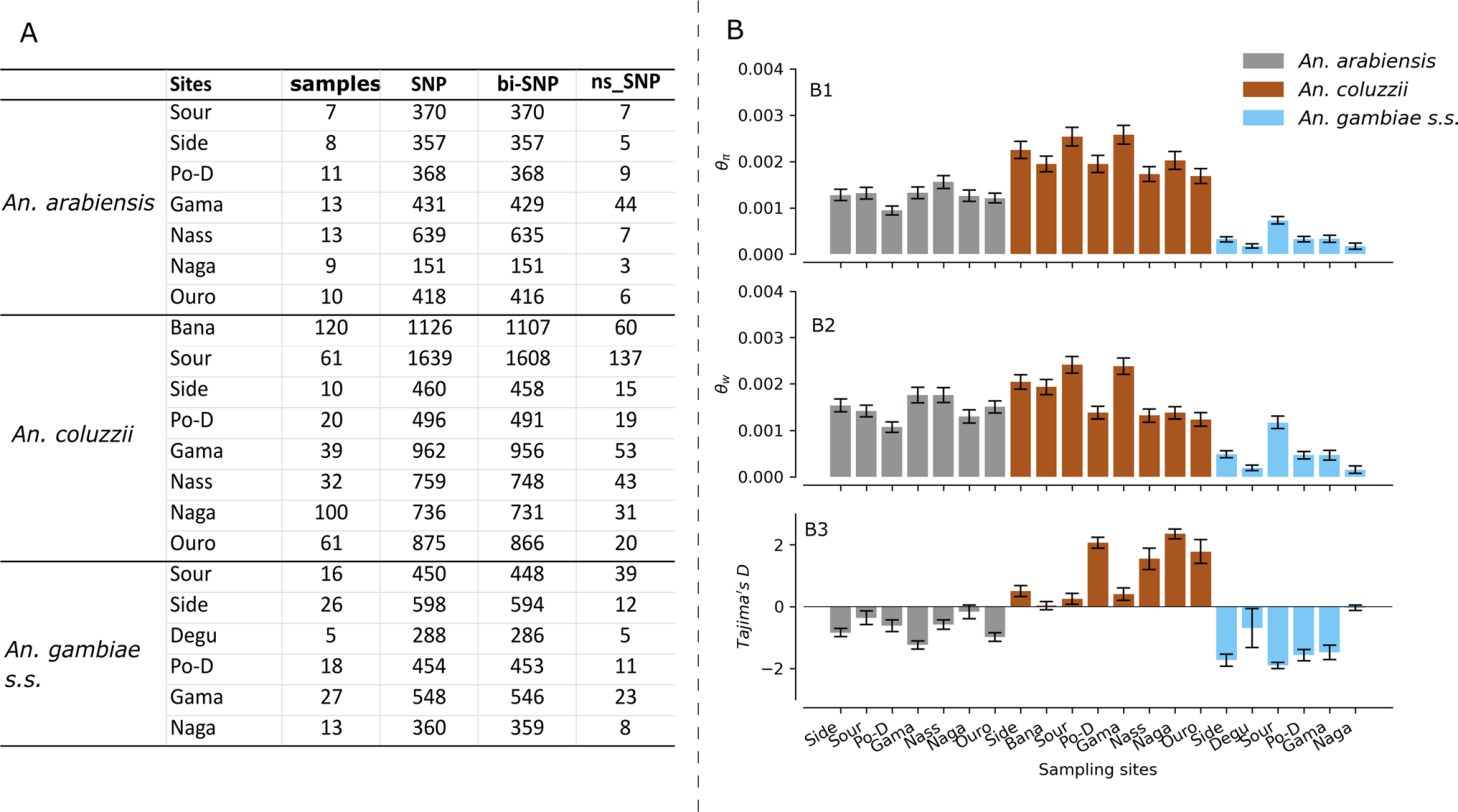
Genetic diversity statistics within the *Vgsc* gene in the *An. gambiae* s.l. populations. Figure 1**A** shows the sample size and the number of segregating sites identified in each population; Fig. 1**B** shows the average of diversity statistics, including the nucleotide diversity (θ_*π*_), the Tajima’s D and the Watterson theta (θ_*w*_) in the *An. gambiae* s.l. populations; Nucleotide diversity measures the mean pairwise nucleotide difference per site among sequences within a population; Watterson theta is an estimator of the population mutation rate based on the number of segregating sites normalized by sample size; Tajima’s D is a neutrality test that compares the genetic diversity based on nucleotide diversity and the segregating sites, with deviations from zero indicating departures from neutral expectations. SNP: Single Nucleotide Polymorphism, bi-SNP: biallelic Single Nucleotide Polymorphism, ns-SNP: Non-Synonymous Single Nucleotide Polymorphism, Bana: Bana Village, Side: Sideradougou, Sour: Souroukoudinga, Po-D: Po-Dongo, Nass: Nassan, Naga: Nagaré, Ouro: Ouro-Hesso, Degu: Deguê-Deguê.

The estimation of the genetic diversity statistics of the *Vgsc* gene revealed a substantial and contrasted diversity between the different *An. gambiae* s.l. populations. Approximately 5121 segregating sites were identified in the *Vgsc* gene within the *An. gambiae* s.l. populations and around 7.18% [368/5121] of these sites having more than two alleles. The variant density (0.07 bp^− 1^) was low in the *Vgsc* gene compared to the whole genome of *An. gambiae*^18^, for which the average was found to be SNP every 2.2 base pairs. Non-synonymous SNPs have been identified within the gene and accounted for 13.18% [675/5121] of the total segregating sites. Figure 1A shows the sample size and the number of segregating sites identified in each population. The number of single nucleotide polymorphisms was variable between the three species with the *An. coluzzii* populations exhibiting the high number of SNPs (3978 SNPs) and the other species *An. gambiae* s.s. and *An. arabiensis* showing 1244 and 1144 SNPs respectively (Fig. 1A). Figure 1B shows the average of diversity statistics, including the nucleotide diversity, the Tajima’s D and the Watterson theta in the *An. gambiae* s.l. populations (Fig. 1B). The overall nucleotide diversity (*θ*_*π*_ *= *0.0021) was higher than those observed in the *An. gambiae* s.s. and *An. arabiensis* populations which were 0.0007 and 0.0017 respectively. In contrast, *An. coluzzii* populations exhibited a slightly higher nucleotide diversity (*θ*_*π*_ = 0.0022) and positive Tajima’s D (*D = *0.953, *θ*_*π*_ = 0.0022, and *θ*_*w*_ *= *0.0018) suggesting an excess of intermediate-frequency alleles in the populations probably due to balancing selection or population structure or decline.

### Kdr mutation allele frequencies

A total of 675 non-synonymous single nucleotide polymorphisms (SNPs) were identified in the vector populations, with frequencies ranging from 0.004167 to 1. Of these, 20 showed frequencies higher than 0.05 (Table S1). The main kdr mutations (*L995F*,* L995S*,* V402L*,* I1527T* and *N1570Y*) associated with pyrethroid resistance were found in the populations at varying frequencies^9^.

**Fig. 2:**
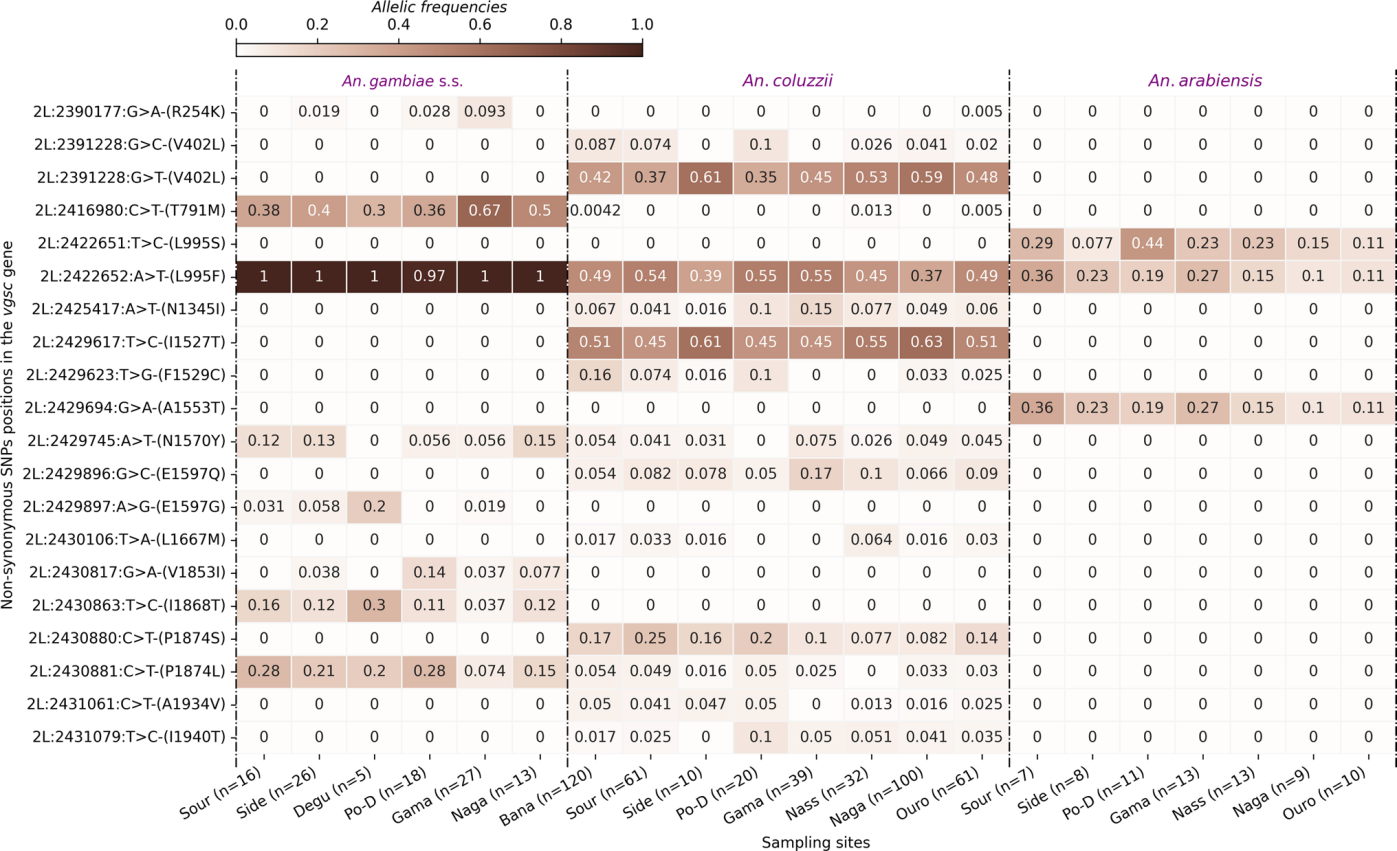
Heatmap showing the distribution of the kdr allele frequencies in the sampling sites. The X‑axis shows the sampling sites. The Y‑axis shows the non‑synonymous SNPs positions in the chromosome 2 L, the nucleotide changes and its corresponding amino acid change. The gradient color bar shows the distribution of the allelic frequencies; Bana: Bana Village, Side: Sideradougou, Sour: Souroukoudinga, Po-D: Po-Dongo, Nass: Nassan, Naga: Nagaré, Ouro: Ouro-Hesso, Degu: Deguê-Deguê.

The SNP *L995F*, formerly known as *L1014F*, is identified in the three populations of *An. gambiae* s.l. at different frequencies. This mutation was found at frequencies reaching fixation in the *An. gambiae* s.s. populations in all the ecological settings of the country suggesting no ecological influences on the spread of this variant (Fig. 2). In *An. coluzzii* populations, the frequencies ranged from 0.37 to 0.55, with the highest frequencies observed in the Sudanian zone, an ecological zone characterized by high pressure of insecticide uses in agriculture such as cotton cultivation. A slight decrease in the frequencies of *L995F* allele was observed in the *An. coluzzii* populations as we moved to the Sahelian zone in the northern part of the country (Fig. 2), especially in Nagaré, Nassan and Ouro-Hesso (Fig. 2). Overall, the results showed a decrease in the *L995F* frequencies in *An. coluzzii* compared to *An. gambiae* s.s. populations where this allele is found at high frequencies (Freq > 0.9).

**Fig. 3:**
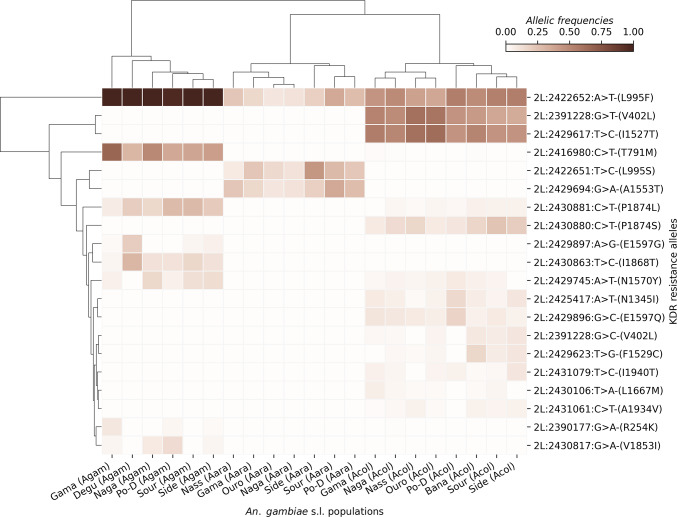
Cluster Map showing the evolutionary landscape and the cooccurrence patterns of the pyrethroid target-site resistance mutations. This figure shows that there is no clear correlation between the kdr mutations and sampling sites or ecological zones. While some of these mutations were shown to occur together in the same populations, there was no association with a specific ecological zone or sampling site. This suggests that there is no ecological segregation linked to pyrethroid resistance in *An. gambiae* s.l. populations in Burkina Faso. The Y‑axis shows the non‑synonymous SNPs positions in the chromosome 2L, the nucleotide changes and its corresponding amino acid change. The gradient color bar shows the allelic frequencies; Side: Sideradougou, Sour: Souroukoudinga, Po-D: Po-Dongo, Nass: Nassan, Naga: Nagaré, Ouro: Ouro-Hesso, Degu: Deguê-Deguê.

The double-mutant *V402L+I1527T* was also observed in the vector populations, specifically in the *An. coluzzii* populations, as observed in previous studies^9,13^. These mutations were observed at high frequencies ranging from 0.37 to 0.63. The *I1527T* is identified in the same populations, following the same trend as the variant *V402L* (Fig. 3). Low frequencies were observed in the Sudanian zone with the populations of Bana (Freq = 0.51), Souroukoudinga (Freq = 0.45), Sideradougou (Freq = 0.61) and Po-Dongo (Freq = 0.45) (Fig. 2). However, the Sahelian population exhibited these mutations at frequencies reaching 0.61 in Nassan and 0.63 in Ouro-Hesso. The *V402L* variant originates from a triallelic locus at position *2L:23,921,228*, where the wild-type nucleotide is G, while the mutant nucleotides are C and T (Fig. 2). These nucleotide changes result in the same amino acid substitution: valine to leucine at position 402 of the *VGSC* protein. These alleles were observed at different frequencies, the *V402L (g > c)* at low frequencies (0-0.087) and the *V402L (g > t)* at frequencies reaching 0.61. Both alleles have been identified in all the sampling sites, except Nassan and Po-Dongo where only *V402L (g > t)* was found. Interestingly, the cumulative frequencies of both alleles are equal to those of the *I1527T*, except the populations of Nagaré and Souroukoudinga where the frequency of *I1527T* is 0.51 and the cumulative frequencies of the *V402L* are 0.44 and 0.505 respectively. These results suggest a potential imbalance in the linkage of the *V402L+I1527T* that were previously identified as linked variants^9,13^.

The analyses also identified other resistant variants, including the *N1570Y* and the *L995S.* The *N1570Y* mutation^14^, was observed in *An. coluzzii* and *An. gambiae* s.s. populations at frequencies up to 0.15. The *L995S* was only observed in the *An. arabiensis* populations, alongside the *L995F*, at frequencies ranging from 0.1 to 0.36 (Fig. 2).

### Genetic background of the kdr alleles

#### Cluster Map

A cluster map was used to explore the evolutionary landscape and the cooccurrence patterns of the pyrethroid target-site resistance mutations in the vector populations. The analysis showed a potential genetic linkage or selective pressure within the *Vgsc* gene, favoring the associative presence of certain variants within each *An. gambiae* s.l. species (Fig. 3). *L995F* was not associated with the same variants across all collection sites, but the populations carrying this SNP also carry other SNPs such as *T791M*,* P1874L*,* N1570Y*,* V1853I*,* E1697G*,* R254K* and *I1868T* at relatively high frequencies in *An. gambiae* s.s. Some mutations were found to be specific to *An. gambiae* s.s., especially the *T791M*,* E1697G*,* I1868T*, and *V1853I* (Fig. 3). *Anopheles coluzzii* showed a different situation with two distinct groups carrying *L995F*,* V402L*,* I1527T*,* P1874S* and other minor alleles at low frequencies. In addition, several mutations including *P1874S*,* N1345I*,* L1667M*,* I1940T*,* A1934V*,* V402L*,* I1527T* and *F1529C*, were found to be specific to *An. coluzzii* populations (Fig. 3). These results are consistent with the observed diversity in *An. coluzzii* populations, suggesting that evolutionary processes such as balancing selection may play a significant role in maintaining these intermediate-frequencies mutations^13^. One group of individuals carrying three SNPs (*L995F*,* L995S* and *A1553T*) at different frequencies have been observed in *An. arabiensis* (Fig. 3). The presence of *L995F* and *L995S* in the same populations confirm previous findings of diverse pyrethroid target-site resistance mechanisms in the same populations^9^. However, the association with the A1553T may suggest potential functional interaction or shared selective advantage associated with the co-occurrence of both *kdr* mutations. While some of these mutations were shown to occur together in the same populations, there was no association with a specific ecological zone or sampling site. This suggests that there is no ecological segregation linked to pyrethroid resistance in *An. gambiae* s.l. populations in Burkina Faso.

#### Genetic background of the kdr alleles

**Fig. 4:**
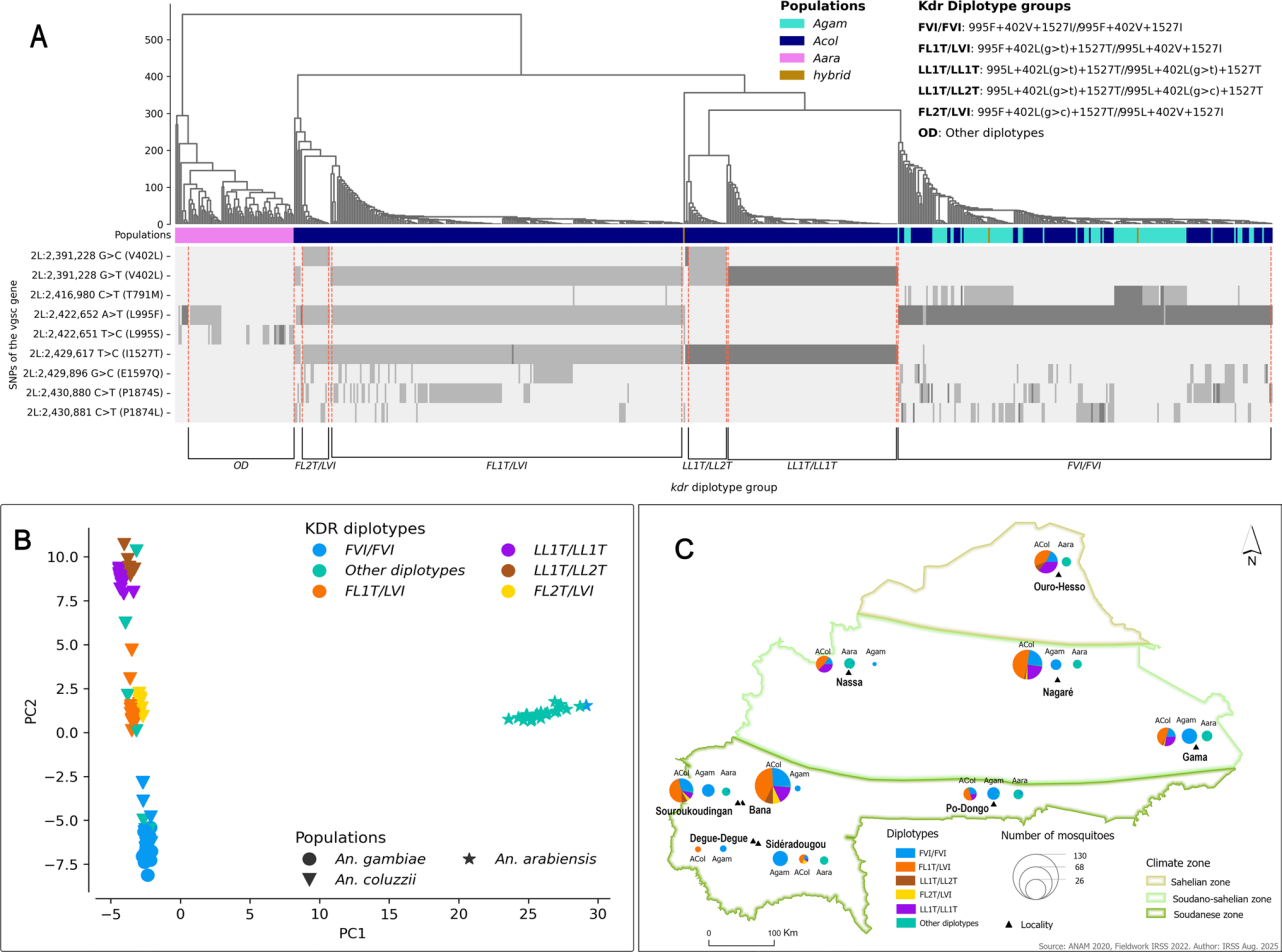
Genetic background of the *kdr* alleles within the *An. gambiae* s.l. population of Burkina Faso. Figure 4**A** shows the diplotype clustering of An. gambiae s.l. populations. Six clusters were identified, each with a different genetic background of kdr alleles. *Anopheles coluzzii* (shown in navy blue) showed five clusters, each with a distinct genetic background; one of these clusters was found to be closely related to *An. gambiae* s.s. populations (shown in turquoise); the individuals in this subpopulation (*FVI/FVI*) were shown to be homozygous for resistant allele *L995F* and the wild-type alleles of the other *kdr* mutations (*V402L* and *I1527T*); the other clusters of *An. coluzzii* consisted of different combinations of the *L995F*, *V402L* and *I1527T* alleles; *An. arabiensis* (shown in violet) was grouped into a distinct cluster comprising combinations of the *L995F* and *L995S* alleles. Figure 4**B** shows the genetic structure of *An. gambiae* s.l. populations driven by the genetic background of the *kdr* mutations. Figure 4**C** shows the geographical distribution and the frequencies of the diplotype groups in Burkina Faso. ***Acol***: *An. coluzzii*, ***Agam***: *An. gambiae s.s*, ***Aara***: *An. arabiensis.* ***Acol***: *An. coluzzii*, ***Agam***: *An. gambiae s.s.*, ***Aara***: *An. arabiensis.* ***A***: *diplotype clustering and the geotypes of each diplotype group;* ***B***: *PCA of the genetic variation within and between the diplotype groups;* ***C***: *spatial distribution and the frequencies of the diplotypes groups found across all sample sites in Burkina Faso*; ***L (995L)***: *wild type allele of L995F;* ***F (995F)***: *mutant allele of L995F;* ***V (402V)***: *wild type allele of V402L;* ***L1 (402L(g > t))***: *first mutant allele of V402L;* ***L2 (402L(g > c))***: *second mutant allele of V402L;* ***I (1527I****): wild type allele of I1527T;* ***T (1527T)***: *mutant allele of I1527T.*

Analyses of the genetic background of the *kdr* alleles revealed complex genotypic variation within the *Vgsc* gene, with six diplotype groups identified in the vector populations (Fig. 4). A diplotype is a pair of haplotypes on homologous chromosomes. It provides a more complete and informative insight into the genetic makeup of an individual organism than a single locus genotype^21^. In this study, the diplotypes were investigated based on the main *kdr* alleles including *L995F*,* L995S*,* V402L(g > t)*, *V402L(g > c)*, and *I1527T* (Fig. 4A). The complex genetic background was mainly observed in the *An. coluzzii* populations, where five diplotype groups were identified. These genotype clusters were identified at different frequencies in the populations, ranging from 0.5 to 0.45. The diplotypes exhibiting various combinations of the double mutant *402L(g > t*,*c)+1527T* were identified within the populations in a homozygous and heterozygous state. These include novel combinations of resistance-associated alleles (*995L+402L(g > t)+1527T/995L+402L(g > c)+1527T (LL1T/LL2T)*,* 995F+402L(g > c)+1527T/995L+402V+1527I (FL2T/LVI) and 995F+402L(g > t)+1527T/995L+402V+1527I (FL1T/LVI)*), suggesting a potential imbalance in the previously observed linkage between both alleles of *402L(g > t*,*c)* and *1527T*^13^, likely due to genetic recombination and gene flow events (Fig. 4A; Table 1).

The PCA grouped these diplotypes into four clusters: two clusters of *An. coluzzii*; one cluster of *An. coluzzii* and *An. gambiae* s.s.; and one cluster of *An. arabiensis* (Fig. 4B). The cluster of *An. coluzzii* and *An. gambiae* s.s. consisted of the homozygote’s individuals carrying the *995F+402V+1527I/995F+402V+1527I* (*FVI/FVI*) genotype. These mosquitoes were shown to carry only the main kdr allele *995F* and the wild type alleles of the other mutations (*402V* and *1527I*). This diplotype group was present in almost all *An. gambiae* s.s. populations at all sampling sites (*freq = 0.991 [112/113]*) (Fig. 4C, Table S2).

The two clusters of *An. coluzzii* consisted of four diplotype groups, one cluster composed of two triple heterozygotes diplotype groups: the *995F+402L(g > t)+1527T/995L+402V+1527I (FL1T/LVI)* clustered with the *995F+402L(g > c)+1527T/995L+402V+1527I (FL2T/LVI)* (Fig. 4A, B). These mosquitoes were shown to carry the main kdr allele *995F*, the *1527T* and both alleles of 402L(g > t,c). This joint clustering suggests that these diplotype groups share similar genetic backgrounds. The diplotype group *FL1T/LVI* was present at all the sampling sites, with frequencies ranging from 0.377 to 0.50 whereas the *FL2T/LVI* was observed at low frequencies ranging from 0.2 to 0.68 in five sites, in the southern part of the country (Fig. 4C).

The last cluster of *An. coluzzii* is also composed of two diplotype groups including the double homozygotes *995L+402L(g > t)+1527T/995L+402L(g > t)+1527T* (*LL1T/LL1T*) clustered with the *995L+402L(g > t)+1527T/995L+402L(g > c)+1527T* (*LL1T/LL2T*). The individuals of the diplotype *LL1T/LL2VT* were shown to include both alleles (*V402L(g > t)* and *V402L(g > c)*) of the *402 L.* One individual mosquito was identified in Souroukoudinga that was found to carry the *1527T* and the *995F* and the wild type allele of the *402L(g > t*,*c)*. The occurrence of these novel genotypes is likely to be the result of ongoing recombination or gene flow events occurring within both triple heterozygotes (*FL1T/LVI* and *FL2T/LVI*) individuals. These groups were found to occur at relatively low frequencies: 0.909–0.375 and 0.20–0.819 for individuals carrying the *FL1T/LVI* and *FL2T/LVI* diplotypes, respectively (Fig. 4C, Table S2). The fourth cluster is distinct from the three others and is composed essentially of *An. arabiensis* populations.

In *An. arabiensis* and *An. gambiae* s.s., the genetic background of *Vgsc* gene was less complex with two clusters, homozygotes *995F/995F (FVI/FVI)* and the other diplotypes (OD) identified within both populations. The homozygotes *995F/995F (FVI/FVI)* were identified at high frequencies reaching 1 in *An. gambiae* s.s. populations (Fig. 4C, Table S2) and carrying the wild type alleles of *L995S*,* V402L* and *I1527T*. Only this cluster was found in *An. gambiae* s.s. with individuals carrying the wild type alleles of the other *kdr* variants (*L995S*,* V402L(g > t*,*c)* and *I1527T*) (Fig. 4A and B). The individuals carrying the diplotype *FVI/FVI*, from either *An. gambiae* s.s. or *An. coluzzii* populations were found to be genetically similar, suggesting a similar genetic background between both species within the *vgsc* gene. The *An. arabiensis* populations exhibited the co-occurrence of the primary kdr alleles *995F* and *995S*, with both variants present in the same mosquito in a heterozygous state. These findings highlight the complex evolutionary dynamics of pyrethroid target-site resistance with the *An. gambiae* s.l. populations.

#### Linkage desequilibrium

Previous studies have identified *402L(g > t*,*c)* and *1527T* as linked alleles, occurring together in the *An. coluzzii* populations. Considering diplotype in this study, we have identified four subpopulations having different combinations of *402L(g > t*,*c)* and *1527T*. The presence of these combinations suggests an imbalance between *402L(g > t*,*c)* and *1527T*. Analysis of the association between the allele *1527T* and both alleles of *402L(g > t*,*c)* showed significant allelic imbalance within the *An. coluzzii* populations. These results indicate a strong linkage disequilibrium (LD) between the allele *1527T* and the allele *402L(g > t)*, with a global *r*^*2*^ of 0.821, suggesting a non-random association between these resistance alleles (Table 1). This strong *LD* was observed in all five villages (Table 1), where the r^2^ was found to range from 0.726 to 1, suggesting a co-selection of both alleles at each site, which can accelerate the development of multiple target-site resistance. Conversely, the analysis revealed a low *LD* (*r² < 0.1*) between *1527T* and *402L(g > c)*, suggesting that both alleles are likely to segregate independently in the vector populations (Table 1). The independent segregation of resistant alleles highlights the need of monitoring each locus separately to understand the prevalence of each variant in a given area. These findings emphasise the importance of analysing the genetic background of insecticide resistance to inform surveillance efforts and help to anticipate the evolutionary dynamics of resistance in mosquito populations.

**Table 1:** Linkage disequilibrium (*r*^*2*^) between the *402L(g > t*,*c)* and *1527T* in the *An. coluzzii* populations. *Strong LD (r*^*2*^ *> 0.6) was observed between 402L(g > t) and 1527T in most An. coluzzii populations*,* indicating strong association between both alleles; However*,* the LD between 402L(g > c) and 1527T was very low (r*^*2*^ *< 0.1)*,* suggesting that both alleles are evolving independently in the vector populations*.

	*n*	LD (*r*^2^)	
		402L(g > c) vs. 1527T	402L(g > t) vs. 1527T
Bana Village	145	0.083	0.726
Souroukoudinga	66	0.091	0.73
Nassan	32	0	1
Sederadougou	10	0.136	0.658
Degue-Degue	4	0	0
Po-Dongo	20	0	1
Gama	39	0.021	0.902
Ouro-Hesso	61	0.025	0.842
Nagare	100	0.02	0.905
*An. coluzzii*	477	0.045	0.821

LD: linkage disequilibrium, *r*^2^: squared correlation coefficient, n: sample size

#### Haplotype network

**Fig. 5:**
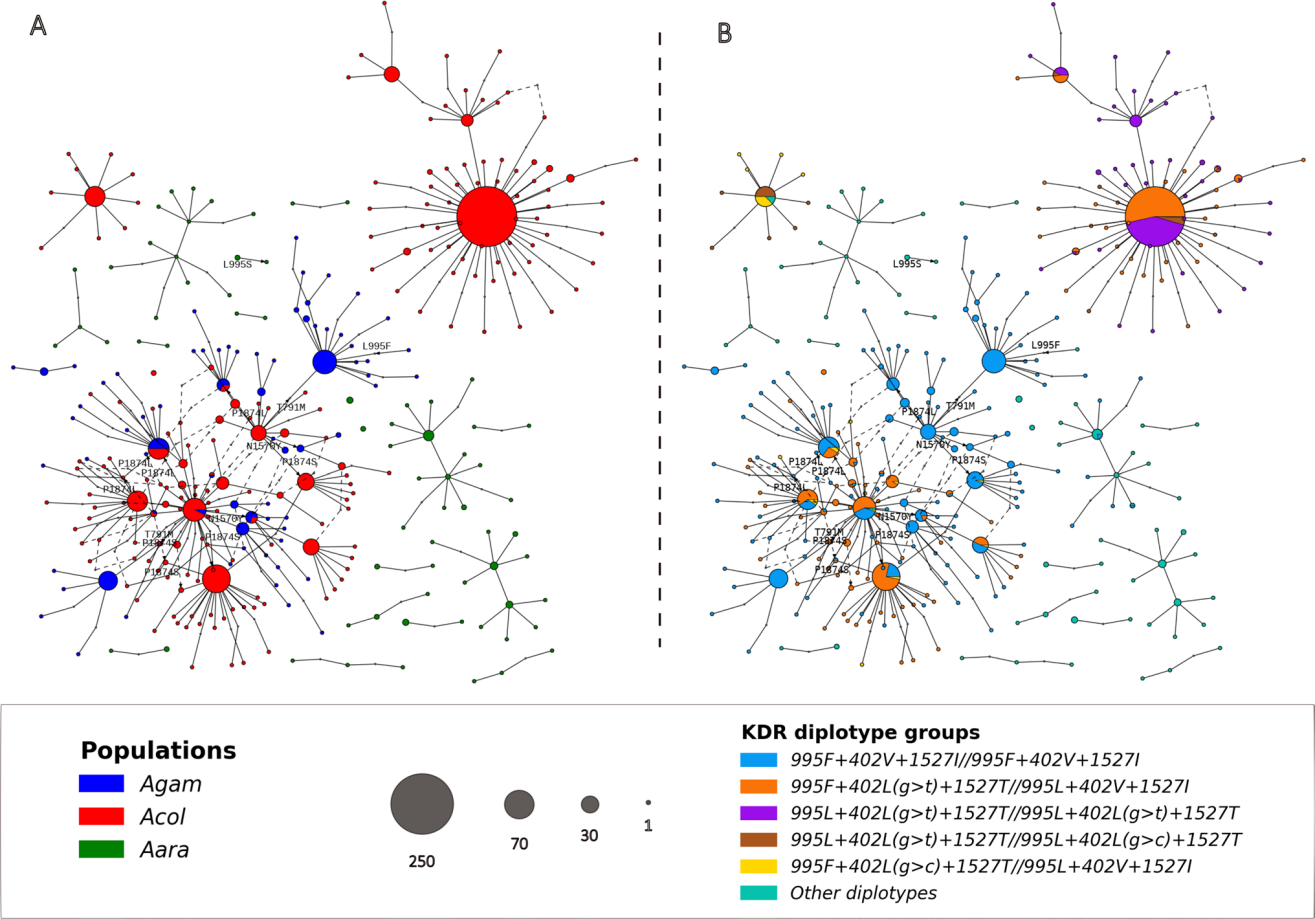
Haplotype network showing the haplotype sharing between *An. gambiae* complex species and the kdr diplotype groups; the circles show the sample size; Fig. 5**A** shows the haplotype sharing and genetic connectivity between *An. gambiae* s.l. populations; it shows three distinct subgroups of *An. coluzzii* populations, one of which subgroup shares haplotypes with *An. gambiae* s.s.; this finding is consistent with that observed in Fig. 4A. Figure 5**B** shows the haplotype sharing and genetic connectivity between the kdr diplotype groups; it shows that *An. coluzzii* populations with the wild-type 995L allele do not share haplotypes with those that have the 995F allele; however, It shows that *An. coluzzii* populations with the 995F allele share haplotypes with each other and with An. gambiae s.s. ***Acol***: *An. coluzzii*, ***Agam***: *An. gambiae s.s.*, ***Aara***: *An. arabiensis.*

The haplotype network showed deep insight into the genetic relationships and evolutionary history of pyrethroid target-site resistance within the *An. gambiae* s.l. population in Burkina Faso (Fig. 5A and B). The network confirms the complex genetic structure observed in the *An. coluzzii* populations. The three clusters of *An. coluzzii* were clearly distinct and no genetic connection was observed between them. The individuals carrying the triple heterozygote genotype (*FL1T/LVI*) were shown to share haplotypes between the diplotype groups identified within the *An. coluzzii* and the *An. gambiae* s.s. populations. The haplotype sharing was observed between the diplotype *FL1T/LVI* and the other diplotypes such as the *FVI/FVI*, the *LL1T/LL1T*, and the *LL1T/LL2T* on one hand; and between the diplotype *FL1T/LVI* and the other diplotypes such as the *FVI/FVI*, the *LL1T/LL1T*, and the *LL1T/LL2T* on other hand (Fig. 5). The connection between the triple heterozygotes and the other diverged diplotypes groups may indicate potential gene flow and/or recombination events occurring in the *An. coluzzii* populations. While haplotype sharing was observed between the diplotype groups of *An. coluzzii*, there was no geographical structure associated with this pattern. This lack of geographical structure suggests a continuous exchange of resistant alleles and haplotypes exchanges across the ecological zones. These results highlight the influence of evolutionary processes on the genetic structure of *An. coluzzii* populations, particularly the emergence of new genotypes through recombination events and the spread of the new emerged diplotype groups across the country through gene flow. In *An. coluzzii* and *An. gambiae* s.s. populations, haplotype sharing within each diplotype group of these populations and no geographical structure was associated with this pattern. These results revealed the complex genetic basis of pyrethroid target site resistance, highlighting the emergence of a novel combination of resistance alleles through recombination or gene flow. They also demonstrated how these medically relevant genotypes can spread rapidly and unhindered across countries, affecting vector control interventions. The identification and monitoring of these new combinations of resistance alleles highlight the importance of the ongoing genomic surveillance of malaria vectors to monitor the local dynamics of resistance alleles that may not be apparent through country-wide global spatial analyses.

### Organophosphate target-site resistance

**Fig. 6:**
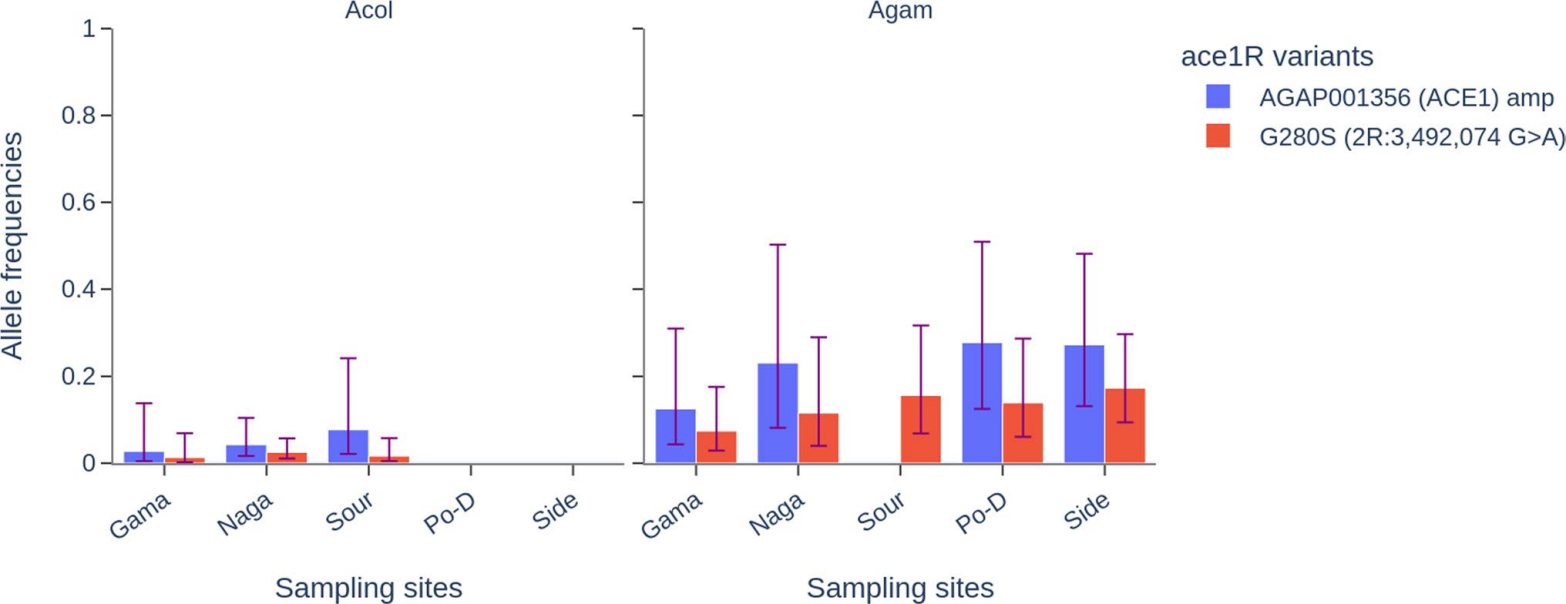
Distribution of the *ace1R-G280S* and *ace1_amp* allele frequencies in the sampling sites. ***Acol***: *An. coluzzii*, ***Agam***: *An. gambiae s.s.*, ***Naga***: *Nagaré*, ***Sour***: *Souroukoudinga*, ***Po-D***: *Po-Dongo*, ***Side***: *Sideradougou*.

Genomic analyses were done in the *Ace1* gene (*2R:3483099-3497400*) to investigate the distribution and the frequencies of the variants involved in organophosphate and carbamate resistance. The analysis identified a total of 4179 segregating sites (about 19.43 % of multiallelic sites) among which 139 SNPs were non-synonymous coding sites (Table S3). These SNPs were found at various frequencies, reaching fixation in some populations. The two main variants associated with organophosphates and carbamates resistance, *ace1R-G280S* and ace1-amp, were identified in the mosquito populations at five sites, including Gama, Nagare, Souroukoudinga, Po-Dongo and Sideradougou (Fig. 6). These variants were found at different frequencies in the *An. coluzzii* and *An. gambiae* s.s. populations. The frequencies of both variants were relatively low, ranging from 0.07 to 0.173 for *ace1R-G280S* and from 0.125 to 0.277 for ace1R-amp. In the *An. coluzzii* populations, these variants were found at low frequencies (freq < 0.05) in three sites, including Gama, Nagare and Souroukoudinga (Fig. 6).

### Metabolic resistance

In *Anopheles* mosquitoes, various gene families, including cytochrome P450 (CYP), carboxylesterases (COEs) and glutathione-S-transferases (GST), have been shown to be involved in the rapid detoxification of insecticides, causing resistance to the insecticides used in public health^22^. Recent investigations have shown that the number of copies of these genes in an individual mosquito may influence the level of expression and contribute to the development of metabolic resistance^23^. We investigated the extent of the copy number variation (CNV) in the *An. gambiae* genome in eight (8) sites in Burkina Faso using statistics based on the depths of WGS coverage. The study identified a total of 142 detoxifying genes (from the COE, GST and CYP families) with at least one CNV in the *An. gambiae* s.l. genome. Of these, 63 showed gene deletion (del) and 122 showed gene amplification (amp). Some genes were found to be deleted in some individuals and amplified in others. The number of detoxifying genes exhibiting copy number variation was variable between chromosomes: 26 were observed on 2L, 50 on 2R, 15 on 3L, 37 on 3R and 14 on the X chromosome (Table S4).

**Fig. 7:**
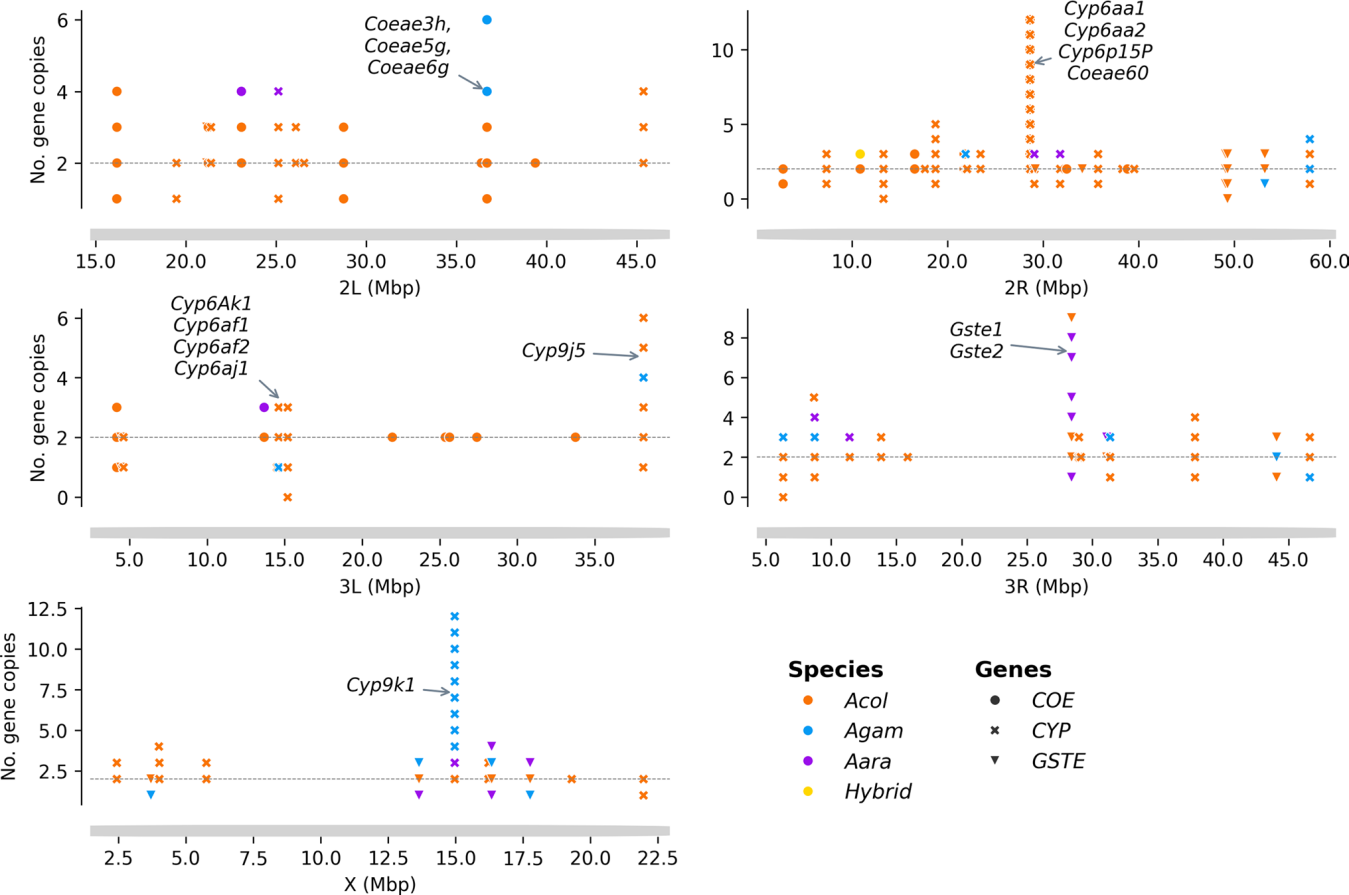
Distribution of the copy number variation of the detoxifying genes within the *An. gambiae* s.l. genome. X-axis shows the genome position; Y-axis shows the number of detoxifying genes copies; the horizontal line indicates the normal copes of the genes while markers upper this line indicates presence of additional copies (gene amplification) and markers below this line indicate deletion of copies (gene deletion) of the corresponding gene; the type of markers represent the different detoxifying genes (point: carboxylesterase,* X filled: cytochrome P450 and triangle_down: glutathione-S-transferases) and the color the An. gambiae complex species; the arrows indicate the position of detoxifying genes that showed a high number of CNVs. CYP: cytochrome P450 genes*,* COE: carboxylesterase genes*,* GST: glutathione-S-transferases genes*,* Acol: An. coluzzii*,* Agam: An. gambiae s.s.*,* Aara: An. arabiensis*.

The analysis identified 25 COE genes showing at least one CNV in the *An. gambiae* s.l. genome, with 16, 5 and 4 genes found on chromosomes 2L, 2R and 3L, respectively. Most of these CNVs were gene amplifications, which were found in 24 of the 25 genes. Almost all of these CNVs (24 gene amplifications and 6 gene deletions) were identified in *An. coluzzii* populations. Few copy number variants were observed in other species, including 15 gene amplifications and two gene deletions in *An. gambiae* s.s., and 11 gene amplifications in *An. arabiensis*. The maximum number of copies was found in the *COEAE60* gene, with up to 12 copies present in a single mosquito (Fig. 7, Table S4). These CNVs were identified at frequencies reaching 84.93% in some mosquito populations. The amplification of AGAP002863 (*COEAE60*) was found at high frequencies from 26.32 to 84.93 in most *An. coluzzii* populations, with frequencies of 84.93%, 73.08% and 64.29% in the populations of Bana, Souroukoudinga and Nassan respectively. The amplification of the gene AGAP005835 (*COEJHE3E*) is found at frequencies of 50.00% and 63.64% in the *An. arabiensis* populations of Po-Dongo and Nassan (Fig. S1).

The analysis also identified copy number variation in the glutathione S-transferases (GST) genes. A total of 26 GST genes exhibited CNVs, of which 16 genes underwent gene deletions and 19 had amplifications. These CNVs were distributed in the mosquito genome as follows, 1 amp on 2L, 6 del and 5 amp on the 2R, 1 del on the 3L, 4 del and 10 amp on the 3R, 5 del and 3 amp on the X chromosome. Two GSTe genes were observed to have a high number of copies and deletions: the AGAP009194 (*GSTE2*) which showed up to 9 copies in *An. coluzzii*, and the AGAP004173 (*GSTD5*), that was completely deleted in *An. coluzzii* and *An. gambae* s.s. populations (Fig. 7, Table S4). The frequencies of these CNVs were variable between the species and the sampling sites with a maximum reaching 100% of some mosquito populations. The deletions of AGAP004173 (*GSTD5*) were found at very high frequencies in the mosquito vector populations, with a frequency of 100% in *An. gambiae* s.s. and *An. arabiensis*, and a frequency of 53.0-71.43% in *An. coluzzii* populations. Most of the GSTE genes showed CNVs at relatively low frequencies, except the AGAP009194 (*GSTE1*) and AGAP009195 (*GSTE2*) which have shown CNVs in 50% of the vector populations of Po-Dongo (Fig. S2).

Copy number variations were also identified in several Cytochrome P450 genes. A total of 91 genes were observed to have CNVs, of which 41 genes showed gene deletions, and 79 genes showed amplifications. These CNVs are distributed throughout the mosquito genome as follows: 5 del and 5 amp on 2L, 16 del and 35 amp on the 2R, 7 del and 6 amp on 3L, 12 del and 25 amp on 3R, 1 del and 8 amp on the X chromosome. The genes, *CYP6AA1*, *CYP6AA2*, *CYP6P15P* and *CYP9K1* were found to have the highest copy number in the genome, while three other genes (*CYP4H25*, *CYP6AF1* and *CYP12F2*) were found to be completely absent from the genome of some mosquitoes. The number of CNVs was variable between the species. *Anopheles coluzzii* was found to have the highest number of genes showing at least one CNV (85 genes of which 35 del and 68 amp). The other species, *An. arabiensis* and *An. gambiae* s.s. also showed CNVs in approximately 66 genes (19 del and 52 amp) and 60 genes (31 del and 42 amp) respectively (Fig. 7, Table S4). These CNVs occurred at variable frequencies in the vector populations, reaching 100% in some mosquito populations. The amplifications in the CYP9K1 gene were found at high frequencies in *An. coluzzii* (8-71.23%) and *An. gambiae* (84.62–100%) populations but at very low frequencies in the *An. arabiensis* populations. In *An. coluzzii*, the high frequencies of CNVs in *CYP9K1* were found in the populations of Bana (~ 71.32%) and Souroukoudinga (~ 42.31%). The amplifications were also found in the CYP6 gene cluster consisting of *CYP6AA1*,* CYP6AA2* and *CYP6P15P* at high frequencies ranging from 26.32% to 96.15% in the *An. coluzzii* populations. The gene deletions were also observed in some genes, especially the *CYP12F2*, the *CYP9M* and *CYP6AF* cluster genes. The deletion in the *CYP12F2* is found in *An. arabiensis* and *An. coluzzii* at frequencies ranging from 67.57% to 90.43%. The CNVs of the *CYP9M* and *CYP6AF* cluster were identified in all the populations, with the frequencies reaching 100% in some populations (Fig. S3).

## Discussion

The increased emergence of insecticide resistance threatens malaria control in sub-Saharan Africa where vector control relies mainly on insecticide-based tools^24^. Recent studies highlighted the constant evolution and adaptation of malaria vectors to environmental changes such as the implementation of vector control tools^25,26^. This situation compromised the sustainability of the current control tools and called urgently for the development of new control strategies^27^. There is a need to monitor the geographical spread and genetic background underlying these variants to inform the implementation of vector control tools. This study showed the country-wide spread and the genetic background of the insecticide resistance variants in Burkina Faso.

Pyrethroid resistance is widespread in Burkina Faso, and this resistance is commonly driven by the association of multiple variants, including target-site, metabolic resistance variants and increased cuticle thickness in the same mosquito species^28,29^. The knockdown-resistant (*kdr*) mutation is one of the main causes of pyrethroid resistance and characterized by mutations occurring in the gene that encodes the insect’s voltage-gated sodium channel, a crucial protein for nerve function^13^. This mutation has been described in most insects, including mosquitoes, flies, beetles, bed bugs etc^30,31^. In *An. gambiae* s.l. mosquitoes, the *kdr-L995F* and *kdr-L995S* mutations are the two most described and are associated with nucleotides change at positions *2L:2,422,652 (A > T)* and *2L:24,226,521 (T > C)*^26^. These variants were identified in this study with high frequencies in all the sampling sites showing the high spread of pyrethroid target-site resistance variants. As observed in previous studies, the double variant *kdr-V402L(g > t*,*c)+ I1527T* is widespread in Burkina Faso, occurring only in the *An. coluzzii* populations^9,32^. These studies identified these variants in the western part of Burkina Faso at evolving frequencies; however, their country-wide presence seriously affects the efficacy of current control tools that were already threatened by the main *kdr* mutations^9,13^.

*Anopheles gambiae* species are genetically connected in Burkina Faso, with a strong gene flow that influences the emergence and the spread of the insecticide resistance variants in the country^32^. However, PCA analyses using the SNPs in the *vgsc* gene revealed three clusters of *An. coluzzii* populations, based on the combination of the alleles *V402L(g > t*,*c)*,* I1527T* and *L995F*. These sub-clusters were found sympatrically within the three ecological zones of the country, suggesting no geographical structure driven by the pyrethroid target-site variants. This sub-clustering provides insights into the genetic and evolutionary dynamic of the *vgsc* gene in shifting malaria vector populations. The emergence of sub-groups with different *kdr* allele combinations within malaria vectors poses significant challenges in the insecticide resistance surveillance in Sub-Saharan Africa. This situation highlights the need for advanced approaches such as whole-genome or amplicon sequencing to accurately track the spread and the evolution of insecticide resistance variants. Other evolutionary events, such as genetic recombination could provide additional layers of complexity in the dynamics of resistance, complicating the monitoring and the prediction of the resistant variant’s dynamics^33^. In this study, novel allele combinations (*FL1T/LVI*, *FL2T/LVI* and *LL1T/LL2T*) were detected and were shown to be carrying the 995F, both alleles of *402L(g > t*,*c)* and the *1527I*. Interestingly, the individuals of the *LL1T/LL2T* were shown to carry both alleles of the of *402L(g > t*,*c)* and clustering with the triple homozygotes *LL1T/LL1T* carrying the *995L*,* 402L(g > t)* and *1527T*. The individuals of the two other groups (*FL1T/LVI*, *FL2T/LVI*) were found to be clustering together and carrying the *995F*, the *402L(g > t*,*c)* and the *1527T*. The *V402L* and *I1527T* were identified in previous studies as a double mutant, occurring together as linked alleles^9,13^. The LD between the alleles *402L(g > c)*,* 402L(g > t)* and *1527T* indicates little or no recombination between *402L(g > t)* and *1527T*, and an independent assortment between the alleles *402L(g > c)* and *1527T* on the other hand. The increased frequency of association between *1527T* and resistant alleles (*402L(g > t*,*c)*) suggests that the combination of *402L(g > t*,*c)* and *1527T* confers additional fitness to *An. coluzzii*. Although the functional role of I1527T has yet to be confirmed, its co-occurrence with other resistance variants (i.e. *402L* and *995F*) may confer a fitness advantage under insecticide pressure. It is crucial to understand the functional implications of the combination of these alleles to predict the resistance dynamics and to design more effective control strategies.

Several strategies have been developed to mitigate the impact of resistance and increase the efficacy of pyrethroid-treated tools. Thus, dual-insecticide treated nets and nets incorporating piperonyl butoxide (PBO) are increasingly being used and scaled up in malaria-endemic countries with the objective to reduce malaria burden^34^. However, while these strategies have shown significant potential in the reduction of malaria burden, their long-term strategies could be compromised by the growing complexity of insecticide resistance^35^. The emergence of multiple insecticide resistance mechanisms, including target-site, metabolic resistance variants and increased cuticle thickness poses major challenges in the sustainability of the current tools^36^. In most areas, the main variants involved in the resistance are unknown due to the increasing polygeny of the resistance mechanisms. Metabolic pyrethroid resistance is commonly driven by high expression of cytochrome P450 gene families to induce a rapid detoxification of pyrethroid insecticide, however mutations and CNVs in these genes can also contribute to increase the resistance level^37^. Previous studies have indicated the presence of multiple copies of these genes in the mosquito genome acting as an enhancer of the rapid detoxification of pyrethroid insecticide^23^. This study identified high frequencies of CNVs in the established genes involved in pyrethroid metabolic resistance, the *Cyp9K1* and the Cyp6 gene families. While gene amplification can increase the expression levels, the gene deletion can act as the opposite, to reduce the expression levels of the genes targeted by PBO and to increase the expression level of the other types of metabolic genes. Our results also showed high frequency of gene deletion (*Cyp9m* and *Cyp6af* gene families) in vector populations^38^. These results highlight the urgent need for sustainable surveillance tools to track the resistance mechanism and inform the implementation of control tools.

Mosquitoes have developed resistance to almost all the insecticides used in public health^39^. The increasing emergence of organophosphate resistance poses a significant threat to vector control efforts, particularly given that these insecticides are being used more frequently in IRS campaigns as an alternative to pyrethroids compounds^40^. The *ace1-G280S* mutation, in the *Ace1* gene encoding acetylcholinesterase, has been associated with the resistance to organophosphates^41^, such as pirimiphos-methyl. While this mutation exhibited low fitness in association with the *kdr* mutation, the amplification (*ace1*^*D*^) of the ace1 gene was shown to solve this cost, making the individual carrying these variants more resistant^20,42^. Both variants were identified in our study, with a spread spectrum limited to the southern and southwestern areas of the country. In previous work, we demonstrated an increasing evolution of *ace1-G280S* and *ace1*^*D*^ in *An. gambaie* s.s. populations, highlighting the rapid spread of both variants in Burkina Faso, a major threat for the efficacy of IRS^9^. Interestingly, the spread of these variants appears to be associated with the agricultural regions of the country where organophosphate compounds are used intensively in crop and cotton cultivation. In the north, where agricultural activity is low, these variants were found at very low frequencies. On the other hand, the increasing emergence and spread of these variants could be associated with the recent introduction of pirimiphos-methyl-based IRS. Indeed, the National Malaria Control Program of Burkina Faso has implemented the pirimiphos-methyl-based IRS in three health districts of the country from 2018 to 2021^43^. This exposure of insecticide, whether from vector control and/or agricultural practices, will play a role in the development and spread of resistance mechanisms, threatening the efficacy of the vector control interventions. This situation requires coordinated action from the public health and agriculture sectors to develop strategies for the cross-sectoral use of insecticides, synchronized resistance monitoring, and the promotion of sustainable agricultural practices.

Successful insecticide resistance management programs require a better and more solid surveillance strategy to understand vector dynamics and evolutionary patterns that could impact the efficacy of control tools. Genomic surveillance appears to offer powerful tools for monitoring the emergence of new resistance variants and understanding the population dynamics and evolutionary phenomena of vectors that could affect the effectiveness of control strategies^26^. The international collaborative initiative the MalariaGEN Vector Observatory (following the *Anopheles gambiae* 1000 genomes (Ag1000G) project) has provided valuable resources for malaria vector genomic surveillance^18,44^. As shown in this study, high-resolution, recently collected, whole genome variation data enables the detection of resistance-linked variants, and tracking the spread and the genetic background underlying the resistance mechanism. While these genomic resources are crucial for understanding the dynamics of resistance and supporting integrated vector management efforts, there is still a significant delay in the ability of vector control programmes to translate complex genomic data into timely, evidence-based decisions and operational strategies. The challenges of genomic surveillance include the complexity of data that requires computing power and specialized expertise to handle and interpret; the lack of standardized mechanism to display complex genomic finding into operational insights for decision-makers and the disconnection between research purpose and operational policy formulation conducting to fragmented decision-making^45^. The data, API and training resources developed by the MalariaGEN Vector Observatory^46^ provide valuable resources for knowledge translation in insecticide resistance surveillance. These resources could be used to develop a genomic surveillance-informed insecticide resistance management strategy as part of the local knowledge translation strategy, ensuring efficient communication of genomic knowledge to relevant stakeholders and decision-makers.

## Conclusion

This study provided insights into the geographical spread and the genetic background of insecticide resistance variants in Burkina Faso. The *kdr* alleles (*L995F*,* L995S*,* V402L*,* I1527T* and *N1570Y*) were widespread within the mosquito populations. The vector populations exhibited a complex genetic background within the *Vgsc* gene, resulting in the emergence of six diplotype groups within the *An. gambiae* s.l. populations. Most of these diplotype groups were identified in *An. coluzzii* populations, including a new genotype resulting from the combination of the *995 F* and *1527T* alleles, which may be due to recombination events. The other species exhibited two diplotype groups, including a diplotype group composed of the homozygotes *995 F*, and the other group composed of different allele combinations including the *995 F* and *995 S* within the *An. gambiae* s.s. and the *An. arabiensis* populations respectively. The results also revealed a country-wide spread of other resistance mechanisms including the copy number variation in the detoxifying genes and the *Ace1* gene variants in the vector populations. The study demonstrated the benefits of genomic surveillance of insecticide resistance in capturing the evolutionary dynamics of resistance mechanisms within the natural vector population, and how these evolutionary processes can drive the population structure of the malaria vectors. Integrating these insights into the national resistance management systems is essential to sustain the effectiveness of the current vector controls, and to inform the development of innovative vector control tools.

### Material and method

#### Mosquito collection

Mosquito samples were collected in eight villages across the ecological zones of Burkina Faso (Fig. 4C). These ecological zones are characterised by distinct climate, vegetation, and land-use patterns that significantly influence the distribution, the abundance, and the behaviour of mosquito populations as well as the dynamic of malaria transmission^3^. The sampling was performed in four villages, including Bana Village (11°14’01"N, 4°28’21"W), Souroukoudinga (*11°14’08"N*,* 4°32’11"W*), Sideradougou (*10°40’41"N*,* 4°15’23"W*) and Po-Dongo (*11°13’08"N*,* 1°01’12"W*) located in the Sudanian zone. This zone is a transitional ecological region with a tropical wet and dry climate and two seasons: a wet season occurring from May to October, with an average annual rainfall of over 1,200 mm, and a dry season from November to April. The second group of villages are located in the Soudano-Sahelian zone (Nagaré (12°55’35"N, 0°08’32"W), Gama (*12°00’11"N*,* 1°45’43"E*) and Nassan (13°01’41"N, 3°00’50"W)). The Soudano-Sahelian zone is an intermediate between the Sudanian and the Sahelian zones, characterized by a semi-arid climate and grassy savanna vegetation with a short rainy season from June to September. The mean annual rainfall in this climate zone is approximately 900 mm. The last village is in the Sahelian zone (Ouro-Hesso (*14°22’28"N*,* 0°07’40"W*)). The Sahelian zone is the northernmost and driest ecological region in Burkina Faso, characterized by low rainfall (< 600 mm), sparse vegetation, and a reliance on pastoralism and facing significant ecological challenges. Malaria transmission is primarily seasonal in Burkina Faso, with the rainy season being the peak period for transmission due to increased mosquito breeding sites^47^.

Mosquito samples were collected in these areas using pyrethroid spray catches (PSC) from September to November of the year 2022. Female and male mosquitoes were collected and subsequently underwent identification to species level using morphological keys (Gillies and Coetzee, 1987) and preserved in 80% alcohol for further analyses.

#### Whole-genome sequencing of *An. gambiae* s.l.

Mosquitoes collected in the different ecological zones of Burkina Faso were sent for whole genome sequencing at the Wellcome Sanger Institute as part of the MalariaGEN Vector Observatory release 3.11. Full details on sample processing for DNA extraction, library preparation, sequencing, data processing and storage are described in the MalariaGEN website^48^.

Briefly, the genome of 665 *An. gambiae* s.l. samples were individually sequenced at high coverage (30 X) using Illumina technology, generating 150 bp paired-end reads. The raw data were processed for quality control, filtering, sex calling and mapping to the AgamP4 reference genome using BWA version 0.7.15 at the MalariaGen resources center. GATK version 3.7-0 were used for the indel realignment through the RealignerTargetCreator and IndelRealigner and the genotypes calling through the UnifedGenotyper. After variant calling, the raw data in FASTQ format and the aligned data in BAM format were stored in the European Nucleotide Archive (ENA, Study Accession n° ERR12776294-ERR12871281). The called variants in zarr formats, including the samples metadata, were stored on Google Cloud and are accessible via the malariagen_data package^49^ or are directly downloadable. Full details about the quality control, raw data alignment, the variants calling, haplotypes phasing and copy number variants identification are available in the MalariaGen/pipelines GitHub repository^48^.

#### Analyses of genetic variation and insecticide resistance

Genetic variation is an essential component of evolutionary adaptation and plays a critical role in the development of insecticide resistance in vector populations. Several variants have been identified in *An. gambiae* s.l. populations and have been implicated in insecticide resistance across sub-Saharan Africa^14,18^. Understanding the evolution and spread of these variants is essential to inform national malaria control programs. Therefore, whole genome data were used to investigate the distribution, genetic diversity, and the evolution of the insecticide resistance variants in Burkina Faso. The analyses were carried out in Jupyterlab^50^, a web-based interactive development environment using the Python language and a wide range of packages including malariagen_data^49^, scikit-allel^51^, matplotlib^52^, etc. These software and packages made it possible to access the data without downloading it and to use the different models for the analysis.

#### Analyses of the spread of pyrethroid resistance

To explore the genetic diversity and the spread of pyrethroid target site resistance variants, we leveraged the dataset of all the SNP and haplotypes called in the *Vgsc* gene. The diversity statistics (segregating sites, nucleotide diversity (*θ*_*π*_), Watterson theta (*θ*_*w*_) and Tajima’s D *(D)*) were estimated in the populations. Single nucleotide polymorphism variation was assessed using the AGAP004707-RD transcript to identify non-synonymous SNPs and estimate their frequencies in the populations. Spatial analyses were performed to map and show the spread of the main variants (*V402L*,* L995F*,* L995S*,* N1570Y* and *I1527T*) involved in the pyrethroid target site resistance across the country. Statistical tests using multiple comparison tests (ANOVA) were used to compare the frequencies of these variants between the locality and the ecological zones. Cluster analyses were performed to investigate the grouping of the main insecticide resistance variants within and between vector populations. A clustermap was performed to visualize this distribution and show the relative link between each variant, the populations and the environment. The non-random association of the kdr alleles was assessed using the r² statistic, which is a standardized measure of linkage disequilibrium (LD). This statistic is computed for each pair of the unphased genotypes using the maximum likelihood estimation^53^.

Multilocus SNP variation and hierarchical clustering analyses were performed to group similar *An. gambiae* s.l. mosquitoes based on their genotype. Hierarchical clustering was performed using scikit-allel and SciPy clusters^54^ to estimate distance between each diplotype cluster and the genetic background of each diplotype group. The individuals of each cluster were then grouped to estimate their frequencies and their spatial distributions across the ecological zones. Diversity statistics and population structure analyses were also performed to compare the genetic dynamics and background of these diplotype groups. Haplotype networks were constructed to explore the evolutionary relationship between the main variants involved in the pyrethroid target site resistance. The haplotype network was built using the median-joining algorithm as implemented in the Ag1000G phase 2 *Vgsc* report^13^ and visualized with the Graphviz library^55^. All the composite figures were built using the Inkscape software^56^.

#### Analyses of the other resistance variants

The analyses were extended to the insecticides used in public health such as organophosphates and carbamates. The SNP and haplotype called in the genomic region of ACE1 were used to explore the dynamics of organophosphate and carbamate resistance. The analyses were performed to estimate the diversity stats, the SNP frequencies and the spatial distribution of the main mutation (*Ace1-G280S*) across the ecological zones. Hierarchical clustering and population structure analyses were conducted to explore the evolutionary relationships and genetic organization of individuals carrying or not carrying this mutation. The dynamics of genetic variants involved in metabolic resistance were investigated in the vector populations. The spread and density of the copy number variation and SNPs were explored in the main gene involved in metabolic resistance.

## Supplementary Information

Below is the link to the electronic supplementary material.

Fig. S1. Heat map showing the CNVs frequencies of the carboxylesterase genes in the An. gambiae s.l. populations of Burkina Faso. The X axis shows the An. gambiae s.l. populations and the sampling sites. The Y axis shows the positions of the carboxylesterase genes in the genomes and the CNV type (amp: gene amplification, del: gene deletion). The gradient color bar shows the distribution of the allelic frequencies. Bana: Bana Village, Side: Sideradougou, Sour: Souroukoudinga, Po-D: Po-Dongo, Nass: Nassan, Naga: Nagaré, Ouro: Ouro-Hesso, Degu: Deguê-Deguê.

Fig. S2. Heat map showing the CNVs frequencies of the glutathione-s-transferase genes in the An. gambiae s.l. populations of Burkina Faso. The X axis shows the An. gambiae s.l. populations and the sampling locations. The Y axis shows the positions of the glutathione‑s‑transferase genes in the genomes and the CNV type (amp: gene amplification, del: gene deletion). The gradient color bar shows the distribution of the allelic frequencies. Bana: Bana Village, Side: Sideradougou, Sour: Souroukoudinga, Po-D: Po-Dongo, Nass: Nassan, Naga: Nagaré, Ouro: Ouro-Hesso, Degu: Deguê-Deguê.

Fig. S3. Heat map showing the CNVs frequencies of the cytochrome P450 genes in the An. gambiae s.l. populations of Burkina Faso. The X axis shows the An. gambiae s.l. populations and the sampling locations. The Y axis shows the positions of the cytochrome p450 genes in the genomes and the CNV type (amp: gene amplification, del: gene deletion). The gradient color bar shows the distribution of the allelic frequencies. Bana: Bana Village, Side: Sideradougou, Sour: Souroukoudinga, Po-D: Po-Dongo, Nass: Nassan, Naga: Nagaré, Ouro: Ouro-Hesso, Degu: Deguê-Deguê.

Table S1. Distribution of the non-synonymous SNPs frequencies of the VGSC gene (2L: 2358158 - 2431617) within An. gambiae s.l. populations in 8 sites in Burkina Faso.

Table S2. Genotype frequencies of the kdr diplotypes in Burkina Faso.

Table S3. Distribution of the non-synonymous SNPs frequencies of the ACE1 gene (2R: 3484107 - 3495790) within An. gambiae s.l. populations in 8 sites in Burkina Faso.

Table S4. Distribution and position of the copy number variation identified in the Anopheles gambiae populations in Burkina Faso.

## Data Availability

Jupyter Notebooks and scripts to reproduce all the analyses, tables and figures are available in the GitHub repository: [https://github.com/mkient/AgamBF-IR-2022.git](https:/github.com/mkient/AgamBF-IR-2022.git) . The SNPs and haplotypes data are available on the homepage of MalariaGEN and can be accessed using the malariagen_data package. The raw sequences in FASTQ format and the aligned sequences in BAM format were stored in the European Nucleotide Archive (ENA, Study Accession n° ERR12776294-ERR12871281).
