# Supplementary figures and images for: Genomic insights into the spread and evolution of insecticide resistance variants in Anopheles gambiae s.l. from Burkina Faso
Source: Sci Rep. 2026 Apr 1;16:12459. doi: 10.1038/s41598-026-45950-y (PMC13083832; doi:10.1038/s41598-026-45950-y)

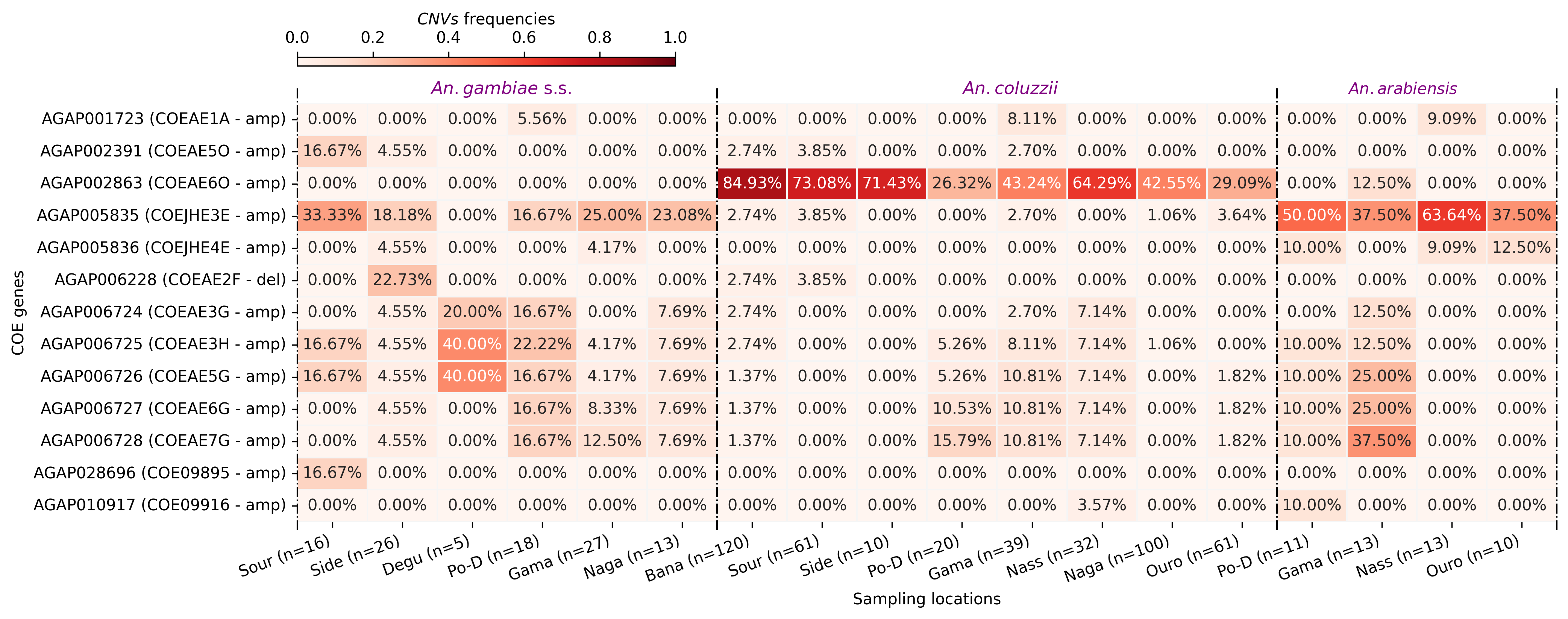

Supplement: Supplementary file 5 — Table S2. Genotype frequencies of the kdr diplotypes in Burkina Faso. [file 41598_2026_45950_MOESM5_ESM.png]

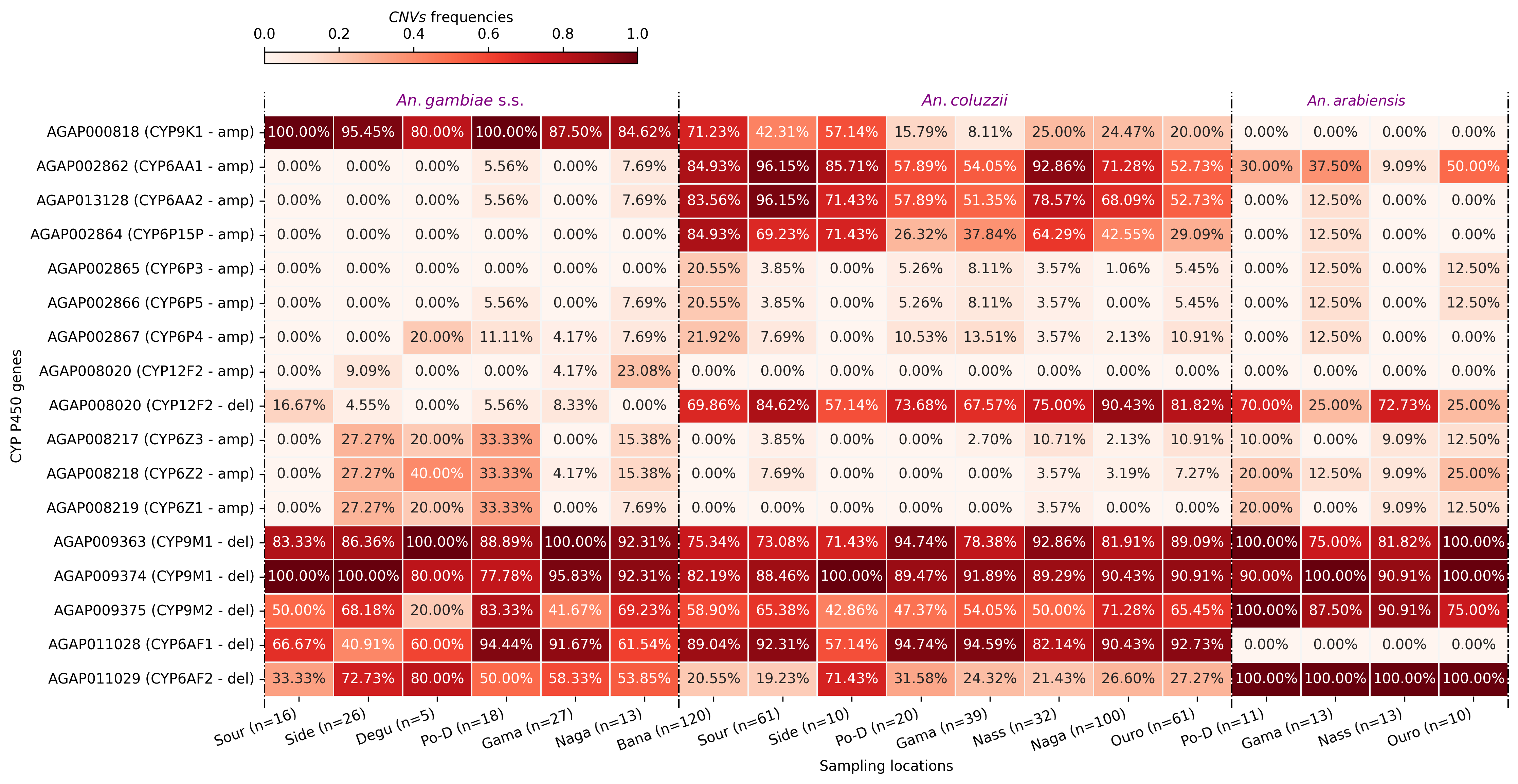

Supplement: Supplementary file 6 — Table S3. Distribution of the non-synonymous SNPs frequencies of the ACE1 gene (2R: 3484107 - 3495790) within An. gambiae s.l. populations in 8 sites in Burkina Faso. [file 41598_2026_45950_MOESM6_ESM.png]

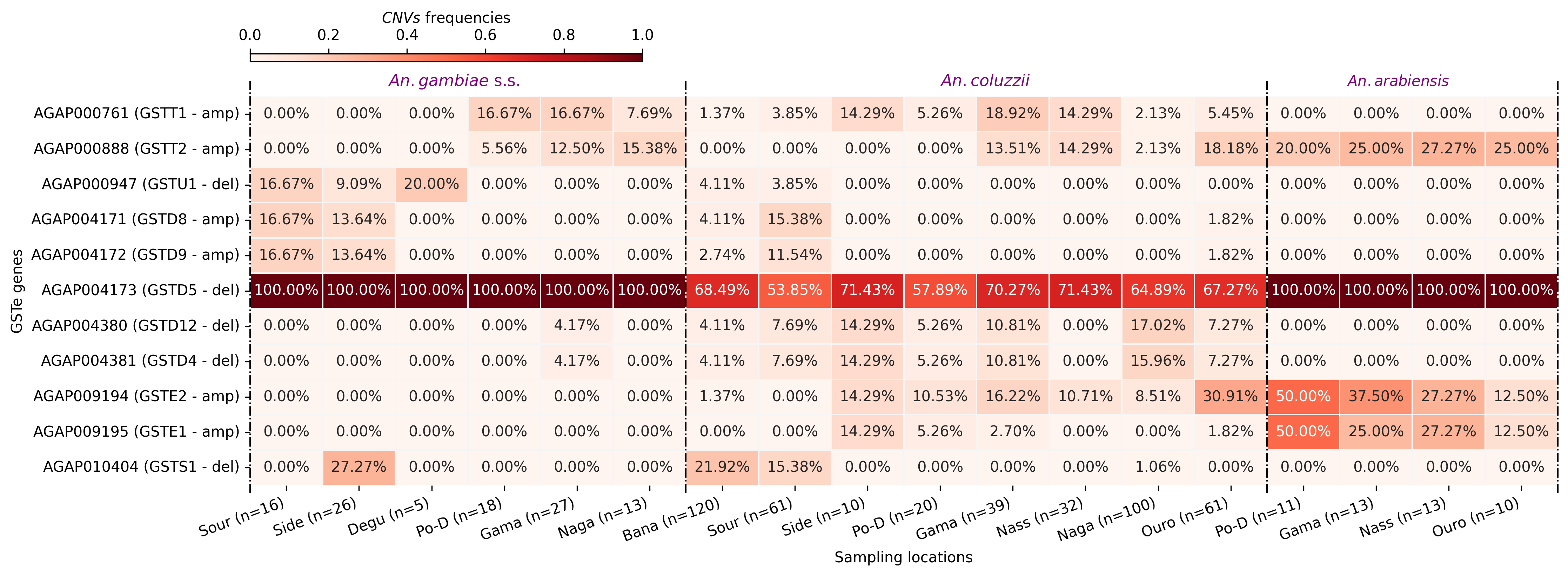

Supplement: Supplementary file 7 — Table S4. Distribution and position of the copy number variation identified in the Anopheles gambiae populations in Burkina Faso. [file 41598_2026_45950_MOESM7_ESM.png]
